# Supplementary material for: Muscle fiber Myc is dispensable for muscle growth and its forced expression severely perturbs homeostasis
Source: Nat Commun. 2025 Apr 3;16:3190. doi: 10.1038/s41467-025-58542-7 (PMC11968879; doi:10.1038/s41467-025-58542-7)
Supplement: Supplementary file 1 — Supplementary Information [file 41467_2025_58542_MOESM1_ESM.pdf]

SUPPLEMENTARY FIGURES

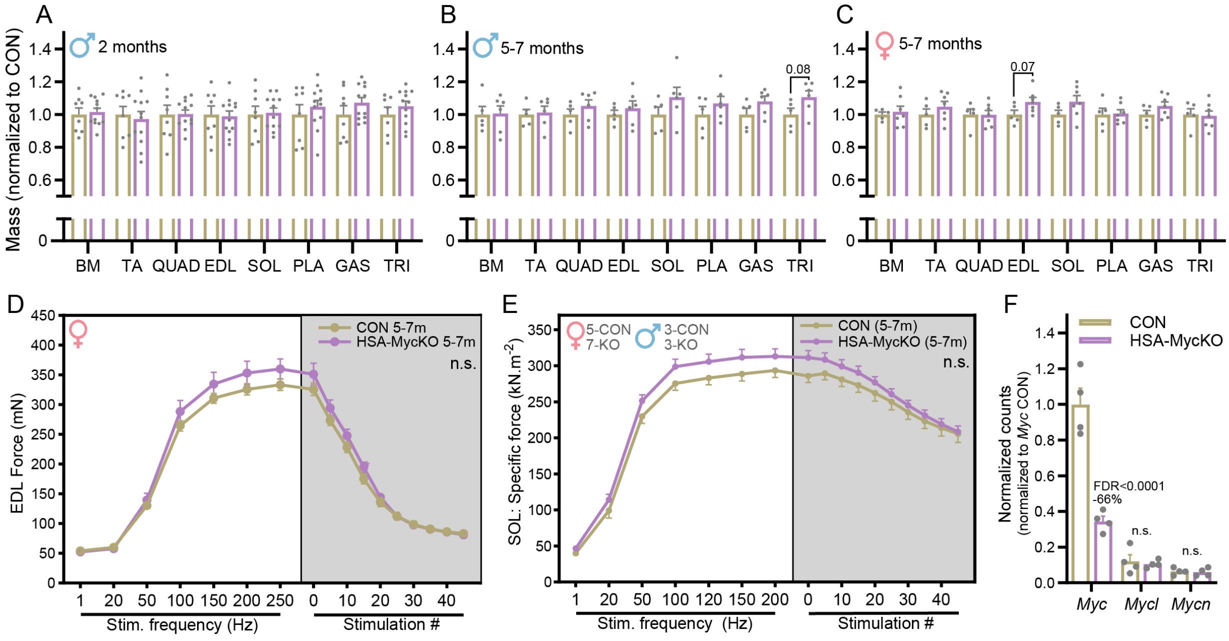

Supplementary Figure 1. Body mass (BM) and *tibialis anterior* (TA), *quadriceps femoris* (QUAD), *extensor digitorum longus* (EDL), *soleus* (SOL), *plantaris* (PLA), *gastrocnemius* (GAS) and *triceps brachii* (TRI) muscle mass, normalized to CON, for **(A)** 2-month-old males (CON, n=8; KO, n=12), **(B)** 5-7-month-old male mice (CON, n=5; KO, n=6) and **(C)** 5-7-month-old female mice (CON, n=5; KO, n=7). Force frequency curve and fatigue response to multiple stimulations expressed as **(D)** absolute force for EDL muscles from female 5-7-month-old mice (CON, n=8; KO, n=7) and **(E)** specific force for SOL muscles from 5-7-month old mice of mixed sex (CON, n=8; KO, n=10). (F) Normalized counts (normalized to *Myc*, CON) from mRNAseq data generated from the GAS muscle of 5- to 7-month-old CON and HSA-MycKO male mice (n=4). Data are presented as mean ± SEM. Students t-test (A-C, F) and Two-way repeated measures ANOVA with Fisher's LSD post-hoc tests (**D-E**) were used to compare between data. For (F), P-values were adjusted for multiple comparisons using the Benjamini-Hochberg (FDR) procedure.

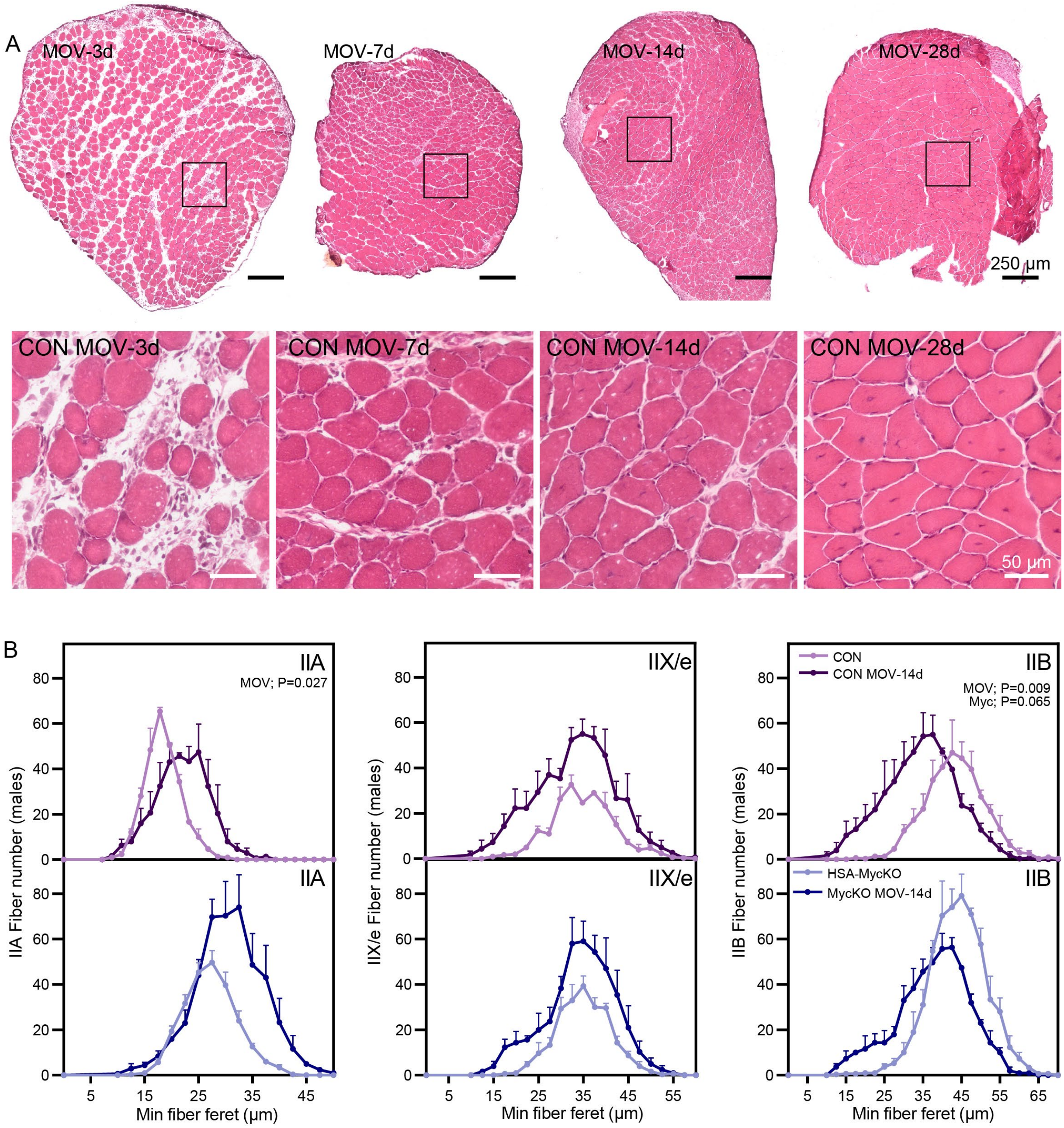

Supplementary Figure 2. (A) Representative haematoxolyin and eosin (H&E) images of *plantaris* (PLA) muscle cross-sections from CON mice after 3, 7, 14 and 28 days of mechanical overload (MOV). **(B)** Type-specific (IIA, IIX, IIB) fiber size distribution normalized to contralateral total fiber number after 14d of MOV in male CON and HSA-MycKO mice (n=3). Data are presented as mean ± SEM. Two-way ANOVAs with Sidak's post hoc test were used to compare median fiber size between conditions.

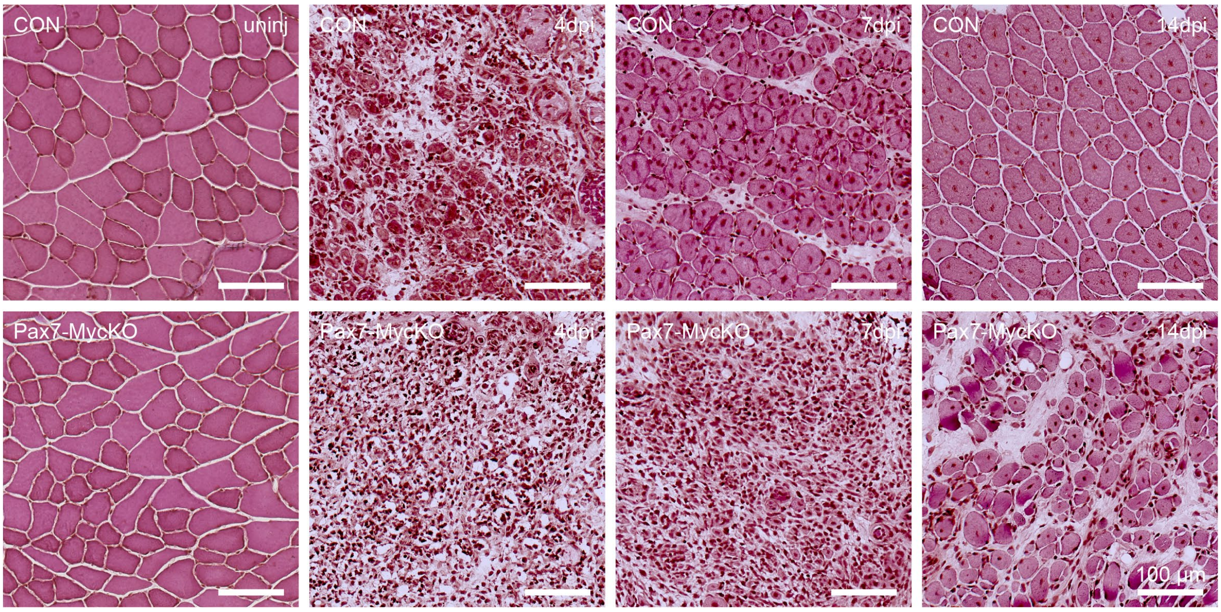

Supplementary Figure 3. Representative haematoxolyin and eosin (H&E) images of TA muscle cross-sections from CON and Pax7-MycKO mice in uninjured muscle and 4, 7 and 14 days after cardiotoxin injection.

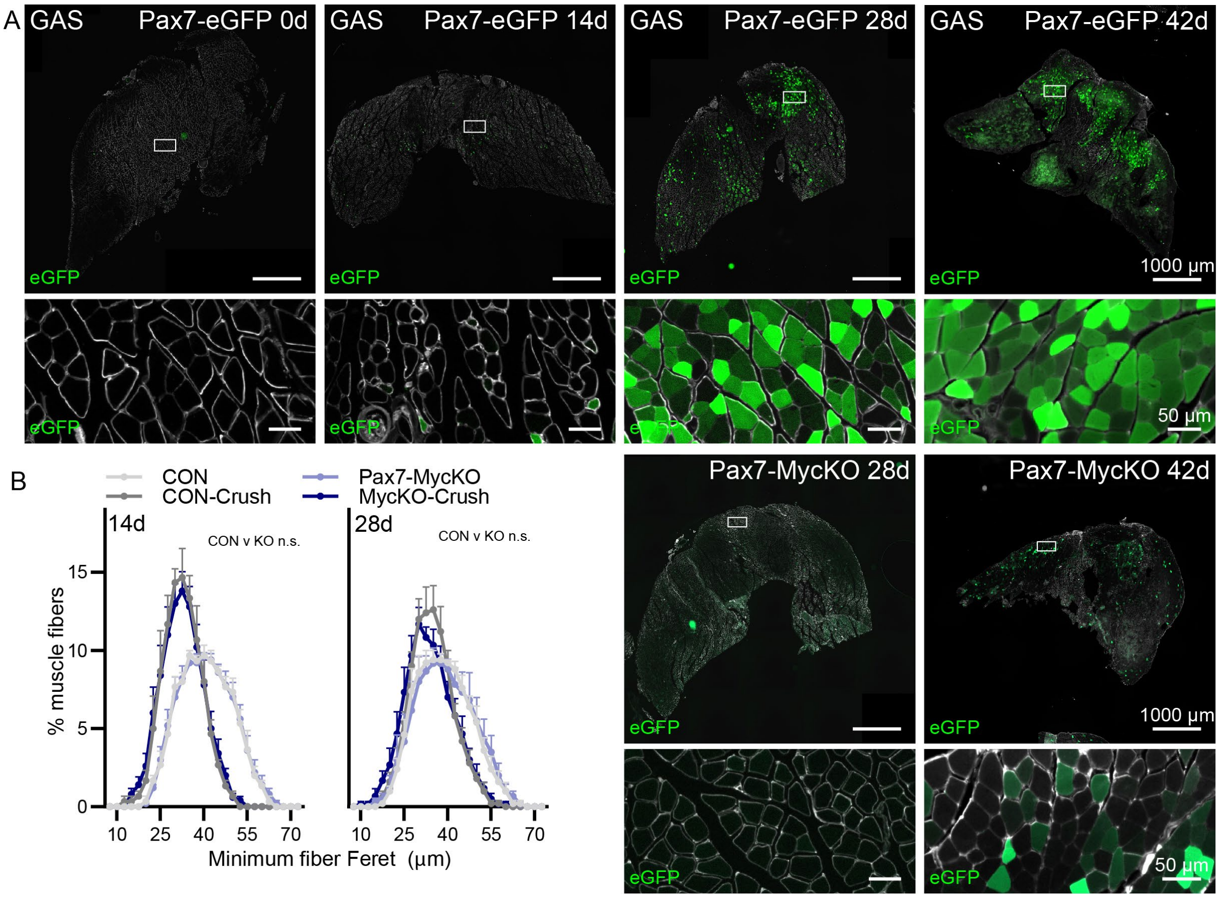

Supplementary Figure 4. (A) Representative images of eGFP+ fibers, indicating fusion of Pax7-eGFP MuSCs in GAS muscle fibers ~6 weeks after tamoxifen treatment in contralateral control muscle (0d) and 14, 28 and 42 days after sciatic nerve crush in Pax7-eGFP mice and 28 and 42 days after nerve crush in Pax7-MycKO mice. Cross-sections are counterstained with laminin (white). **(B)** Fiber size distribution in TA muscle from 14 and 28 days after nerve crush along with their contralateral controls for CON (14d, n=3; 28d, n=5) and Pax7-MycKO (14d, n=5; 28d, n=6) mice. Data are presented as mean ± SEM. Two-way ANOVAs with Sidak's post hoc test were used to compare between conditions.

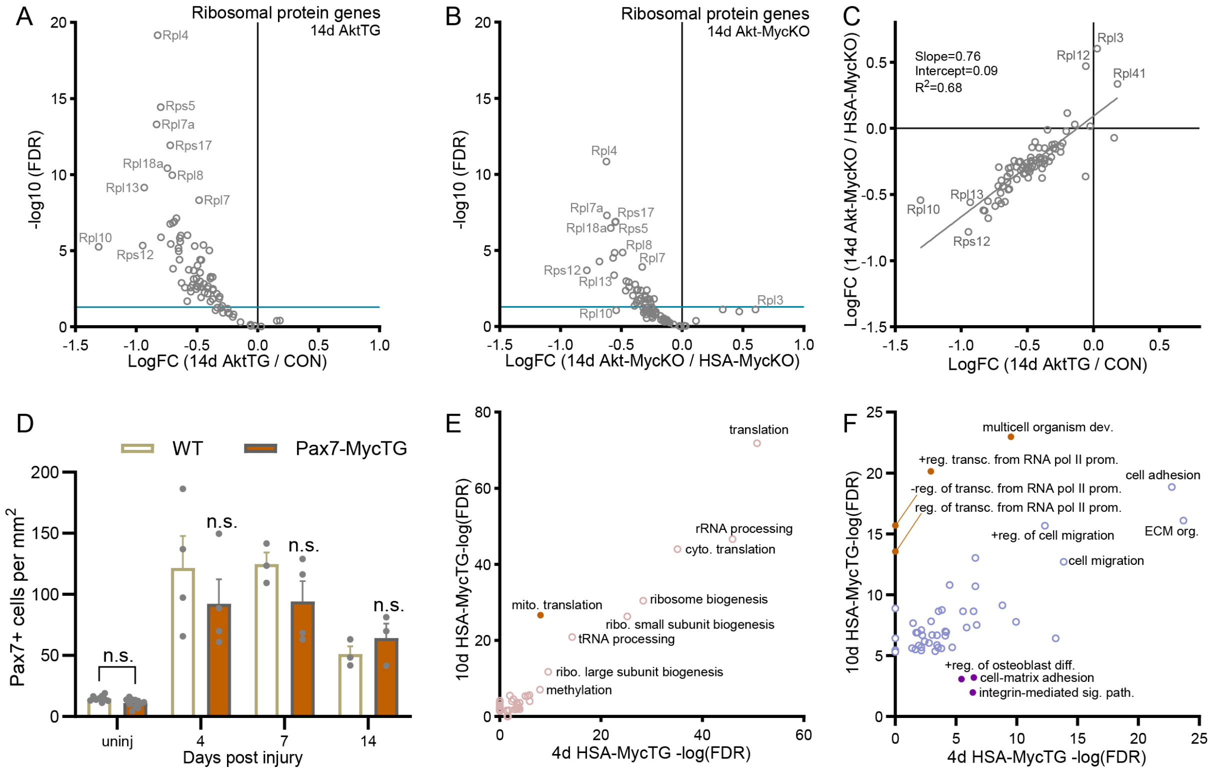

Supplementary Figure 5. Scatterplot of log-fold change (LogFC) and -log10 adjusted P value for all ribosomal protein genes in RNAseq data for comparisons between **(A)** 14d AktTG (n=4) and CON (n=4) and **(B)** 14d Akt-MycKO (n=4) and HSA-MycKO (n=4) mice. **(C)** Scatterplot and regression analysis comparing log-fold changes for all ribosomal protein genes in RNAseq data for 14d AktTG/CON and Akt-MycKO/HSA-MycKO. **(D)** Pax7+ cell number normalized to cross-sectional area in TA muscle sections, in uninjured muscle and 4, 7 and 14 days after cardiotoxin (CTX) injection in CON (n=9, 4, 3, 3, respectively) and Pax7-MycTG (n=11, 4, 4, 3, respectively) mice. **(E)** Upregulated and **(F)** downregulated (right) gene ontology (GO) terms (Biological process) enriched in either 4d (x-axis) or 10d (y-axis) HSA-MycTG (n=4) mice compared to CON (n=7) mice. Top GO terms are labelled, while terms more prominently represented in 4d and 10d HSA-MycTG are shown in purple and orange, respectively. Data are presented as mean ± SEM. Two-way ANOVAs with Sidak's post hoc test were used to compare between conditions.
