# Supplementary material for: Muscle fiber Myc is dispensable for muscle growth and its forced expression severely perturbs homeostasis
Source: Nat Commun. 2025 Apr 3;16:3190. doi: 10.1038/s41467-025-58542-7 (PMC11968879; doi:10.1038/s41467-025-58542-7)
Supplement: Supplementary file 2 — Reporting Summary [file 41467_2025_58542_MOESM2_ESM.pdf]

nature portfolio

Corresponding author(s): Markus A. Rüegg and Daniel J. Ham

Last updated by author(s): 27/2/25

Reporting Summary

Nature Portfolio wishes to improve the reproducibility of the work that we publish. This form provides structure for consistency and transparency in reporting. For further information on Nature Portfolio policies, see our Editorial Policies and the Editorial Policy Checklist.

Statistics

For all statistical analyses, confirm that the following items are present in the figure legend, table legend, main text, or Methods section.

n/a	Confirmed	
☐	☒	The exact sample size (*n*) for each experimental group/condition, given as a discrete number and unit of measurement
☒	☐	A statement on whether measurements were taken from distinct samples or whether the same sample was measured repeatedly
☐	☒	The statistical test(s) used AND whether they are one- or two-sided<br>*Only common tests should be described solely by name; describe more complex techniques in the Methods section.*
☒	☐	A description of all covariates tested
☐	☒	A description of any assumptions or corrections, such as tests of normality and adjustment for multiple comparisons
☐	☒	A full description of the statistical parameters including central tendency (e.g. means) or other basic estimates (e.g. regression coefficient) AND variation (e.g. standard deviation) or associated estimates of uncertainty (e.g. confidence intervals)
☐	☒	For null hypothesis testing, the test statistic (e.g. *F*, *t*, *r*) with confidence intervals, effect sizes, degrees of freedom and *P* value noted<br>*Give P values as exact values whenever suitable.*
☒	☐	For Bayesian analysis, information on the choice of priors and Markov chain Monte Carlo settings
☒	☐	For hierarchical and complex designs, identification of the appropriate level for tests and full reporting of outcomes
☒	☐	Estimates of effect sizes (e.g. Cohen's *d*, Pearson's *r*), indicating how they were calculated

Our web collection on statistics for biologists contains articles on many of the points above.

Software and code

Policy information about availability of computer code

Data collection	FASTQ files were aligned to the indexed mouse transcriptome mm10 using Salmon (version 1.1.0) with the flags validateMappings, seqBias and gcBias. Output quant.sf files from all samples were imported into R (version 4.1.2) using tximeta (Bioconductor). Code is available upon request from Dr. Daniel Ham (dan.ham@unibas.ch). RTqPCR Primers were designed using Geneious®10 software and specificity confirmed by the Basic Local Alignment Search Tool (BLAST). Potential hairpin formation, complementarity and self-annealing sites were verified to be negative by OligoCalc. Westerm Blot protein abundance was quantified using FusionCapt Advance (Vilber).
Data analysis	RNAseq data were analysed using the Bioconductor package DESeq2

For manuscripts utilizing custom algorithms or software that are central to the research but not yet described in published literature, software must be made available to editors and reviewers. We strongly encourage code deposition in a community repository (e.g. GitHub). See the Nature Portfolio guidelines for submitting code & software for further information.

Data

Policy information about availability of data

All manuscripts must include a data availability statement. This statement should provide the following information, where applicable:

- Accession codes, unique identifiers, or web links for publicly available datasets
- A description of any restrictions on data availability
- For clinical datasets or third party data, please ensure that the statement adheres to our policy

All mRNA-Seq data sets are available at the Gene Expression Omnibus (GEO) [85] under the accession numbers GSE287999 [https://www.ncbi.nlm.nih.gov/geo/query/acc.cgi?acc=GSE287999] (HSA-MycKO), GSE288001 [https://www.ncbi.nlm.nih.gov/geo/query/acc.cgi?acc=GSE288001] (HSA-MycTG) and GSE287997 [https://www.ncbi.nlm.nih.gov/geo/query/acc.cgi?acc=GSE287997] (Akt-MycKO).

Research involving human participants, their data, or biological material

Policy information about studies with human participants or human data. See also policy information about sex, gender (identity/presentation), and sexual orientation and race, ethnicity and racism.

Reporting on sex and gender	N/A
Reporting on race, ethnicity, or other socially relevant groupings	N/A
Population characteristics	N/A
Recruitment	N/A
Ethics oversight	N/A

Note that full information on the approval of the study protocol must also be provided in the manuscript.

Field-specific reporting

Please select the one below that is the best fit for your research. If you are not sure, read the appropriate sections before making your selection.

☒ Life sciences ☐ Behavioural & social sciences ☐ Ecological, evolutionary & environmental sciences

For a reference copy of the document with all sections, see nature.com/documents/nr-reporting-summary-flat.pdf

Life sciences study design

All studies must disclose on these points even when the disclosure is negative.

Sample size	Sample sizes were based on previous experience in the lab working with mice (e.g. Ham, Nature Communications, 2022) along with previous sample size estimation analyses (ClinCalc) indicating that 7 per group is sufficient to detect a difference between two groups of at least 15% (Power = 80%; alpha = 0.05) for measures such as in vitro muscle force that have a typical standard deviation around 10%.
Data exclusions	A single outlier in the Akt-MycKO 3 day group was identified in mRNA sequencing analysis and removed from further analysis based on a leg injury noted during dissection.
Replication	Results were reproducible between experiments. All attempts at replication were successful.
Randomization	Groups were assigned based on genotype.
Blinding	In vitro measurements of muscle force were blinded for genotype. Due to technical/researcher limitations and in some cases, obvious phenotypes, blinding was not performed for all other experiments.

Reporting for specific materials, systems and methods

We require information from authors about some types of materials, experimental systems and methods used in many studies. Here, indicate whether each material, system or method listed is relevant to your study. If you are not sure if a list item applies to your research, read the appropriate section before selecting a response.

Materials & experimental systems

n/a	Involved in the study
☐	☒ Antibodies
☒	☐ Eukaryotic cell lines
☒	☐ Palaeontology and archaeology
☐	☒ Animals and other organisms
☒	☐ Clinical data
☒	☐ Dual use research of concern
☒	☐ Plants

Methods

n/a	Involved in the study
☒	☐ ChIP-seq
☒	☐ Flow cytometry
☒	☐ MRI-based neuroimaging

Antibodies

Antibodies used

Myosin 7 mouse IgG2b monoclonal (IHC: 1:50), DSHB BA-D5
Myosin 2 mouse IgG1 monoclonal (IHC: 1:200), DSHB SC-71
Myosin 4 mouse IgM monoclonal (IHC: 1:50), DSHB BF-F3
Myosin 3 mouse IgG1 monoclonal (IHC; 1:00), DSHB F1.652
laminin β1γ1 rabbit IgG polyclonal (IHC; 1:200), Sigma L9393
Pax7 mouse IgG1 monoclonal (IHC, 1:20), DSHB
Myogenin rabbit IgG monoclonal (IHC, 1:400), Abcam Ab124800
laminin β1 rat IgG1 monoclonal (IHC, 1:100), Invitrogen MA5-14657
Ki67 rabbit IgG polyclonal (IHC, 1:200), Abcam Ab15580
GFP chicken IgG polyclonal (IHC, 1:200), Thermo Scientific A10262
Myc (D84C12) Rabbit IgG monoclonal (WB, 1:1000), Cell Signaling 5605
Rps6 (5G10) Rabbit IgG monoclonal (WB, 1:1000), Cell Signaling 2217S
Rps14 Rabbit IgG polyclonal (WB, 1:1000), Proteintech 16683-1-AP
α-actinin mouse IgG1 monoclonal (WB: 1:1000), Sigma A7732
Goat anti mouse polyclonal IgG1 Alexa568 (IHC, 1:100), Invitrogen A-21124
Goat anti mouse polyclonal IgM Alexa488 (IHC, 1:100), Invitrogen A-21042
Donkey anti rabbit polyclonal IgG Alexa647 (IHC, 1:200), Jackson 711-605-152
Goat anti mouse polyclonal IgG2b DyLight 405 (IHC, 1:50), Jackson 115-475-207
Goat anti mouse polyclonal IgG1 Cy3 (IHC, 1:300-400), Jackson 115-165-205
Goat anti rabbit polyclonal IgG Alexa 488 (IHC, 1:200), Invitrogen 11034
Goat anti mouse IgG polyclonal Alexa 647 (IHC, 1:200), Invitrogen 21235
Goat anti rat polyclonal IgG Cy3 (IHC, 1:500), Jackson 112-165-143
Goat anti rabbit polyclonal IgG Cy5 (IHC, 1:300), Jackson 111-175-144
Goat anti chicken polyclonal IgG Alexa488 (IHC, 1:300), Jackson 103-545-155

Validation

Myosin 7 mouse IgG2b monoclonal (IHC: 1:50), DSHB BA-D5, https://dshb.biology.uiowa.edu/BA-D5
Myosin 2 mouse IgG1 monoclonal (IHC: 1:200), DSHB SC-71, https://dshb.biology.uiowa.edu/SC-71
Myosin 4 mouse IgM monoclonal (IHC: 1:50), DSHB BF-F3, https://dshb.biology.uiowa.edu/BF-F3
Myosin 3 mouse IgG1 monoclonal (IHC; 1:00), DSHB F1.652, https://dshb.biology.uiowa.edu/F1-652
laminin β1γ1 rabbit IgG polyclonal (IHC; 1:200), Sigma L9393, https://www.sigmaaldrich.com/CH/de/product/sigma/l9393
Pax7 mouse IgG1 monoclonal (IHC, 1:20), DSHB, https://dshb.biology.uiowa.edu/PAX7
Myogenin rabbit IgG monoclonal (IHC, 1:400), Abcam Ab124800, https://www.abcam.com/en-us/products/primary-antibodies/myogenin-antibody-epr4789-ab124800
laminin β1 rat IgG1 monoclonal (IHC, 1:100), Invitrogen MA5-14657, https://www.citeab.com/antibodies/90026-ma5-14657-laminin-beta-1-monoclonal-antibody-lt3
Ki67 rabbit IgG polyclonal (IHC, 1:200), Abcam Ab15580, https://www.abcam.com/en-us/products/primary-antibodies/ki67-antibody-ab15580
GFP chicken IgG polyclonal (IHC, 1:200), Thermo Scientific A10262, https://www.thermofisher.com/antibody/product/GFP-Antibody-Polyclonal/A10262
Myc (D84C12) Rabbit IgG monoclonal (WB, 1:1000), Cell Signaling 5605, https://www.cellsignal.com/products/primary-antibodies/c-myc-d84c12-rabbit-mab/5605
Rps6 (5G10) Rabbit IgG monoclonal (WB, 1:1000), Cell Signaling 2217S, https://www.cellsignal.com/products/primary-antibodies/s6-ribosomal-protein-5g10-rabbit-mab/2217
Rps14 Rabbit IgG polyclonal (WB, 1:1000), Proteintech 16683-1-AP, https://www.ptglab.com/products/RPS14-Antibody-16683-1-AP.htm
α-actinin mouse IgG1 monoclonal (WB: 1:1000), Sigma A7732, https://www.sigmaaldrich.com/CH/de/product/sigma/a7732
Goat anti mouse polyclonal IgG1 Alexa568 (IHC, 1:100), Invitrogen A-21124, https://www.thermofisher.com/antibody/product/Goat-anti-Mouse-IgG1-Cross-Adsorbed-Secondary-Antibody-Polyclonal/A-21124
Goat anti mouse polyclonal IgM Alexa488 (IHC, 1:100), Invitrogen A-21042, https://www.thermofisher.com/antibody/product/Goat-anti-Mouse-IgM-Heavy-chain-Cross-Adsorbed-Secondary-Antibody-Polyclonal/A-21042
Donkey anti rabbit polyclonal IgG Alexa647 (IHC, 1:200), Jackson 711-605-152, https://www.jacksonimmuno.com/catalog/products/711-605-152/Donkey-Rabbit-IgG-HL-Alexa-Fluor-647
Goat anti mouse polyclonal IgG2b DyLight 405 (IHC, 1:50), Jackson 115-475-207, https://www.jacksonimmuno.com/catalog/products/115-475-207
Goat anti mouse polyclonal IgG1 Cy3 (IHC, 1:300-400), Jackson 115-165-205, https://www.jacksonimmuno.com/catalog/products/115-165-205
Goat anti rabbit polyclonal IgG Alexa 488 (IHC, 1:200), Invitrogen 11034, https://www.thermofisher.com/antibody/product/Goat-anti-Rabbit-IgG-H-L-Highly-Cross-Adsorbed-Secondary-Antibody-Polyclonal/A-11034

Goat anti mouse IgG polyclonal Alexa 647 (IHC, 1:200), Invitrogen 21235, https://www.thermofisher.com/antibody/product/Goat-anti-Mouse-IgG-H-L-Cross-Adsorbed-Secondary-Antibody-Polyclonal/A-21235
Goat anti rat polyclonal IgG Cy3 (IHC, 1:500), Jackson 112-165-143, https://www.jacksonimmuno.com/catalog/products/112-165-143
Goat anti rabbit polyclonal IgG Cy5 (IHC, 1:300), Jackson 111-175-144, https://www.jacksonimmuno.com/catalog/products/111-175-144
Goat anti chicken polyclonal IgG Alexa488 (IHC, 1:300), Jackson 103-545-155, https://www.jacksonimmuno.com/catalog/products/103-545-155

Animals and other research organisms

Policy information about studies involving animals; ARRIVE guidelines recommended for reporting animal research, and Sex and Gender in Research

Laboratory animals	HSA-MycKO, Pax7-MycKO, HSA-MycTG, Pax7-MycTG, Akt-TG and Akt-MycKO mice and their respective controls were bred on a C57BL/6JRj background. Mice were kept on a 12 hr light-dark cycle (6 am to 6 pm) at 22°C (range 20-24°C) and 55% (range 45-65%) relative humidity.
Wild animals	This study did not involve wild animals
Reporting on sex	Both female and male mice were used throughout. Numbers are indicated within the figure/figure legend.
Field-collected samples	This study did not involve field-collected samples
Ethics oversight	All experiments were approved by the regional animal ethics Committee of Basel-Stadt, Switzerland.

Note that full information on the approval of the study protocol must also be provided in the manuscript.

Plants

Seed stocks	N/A
Novel plant genotypes	N/A
Authentication	N/A
